# Editorial: Value-based healthcare in oncology

**DOI:** 10.3389/fpubh.2023.1274409

**Published:** 2023-09-13

**Authors:** Antonio Giulio de Belvis

**Affiliations:** Fondazione Policlinico Universitario “A. Gemelli”, Università Cattolica del Sacro Cuore, Italian National Institute of Care and Research (IRCCS), Italian National Scientific Association of Quality on Health and Social Care (ASIQUAS) National Board, Rome, Italy

**Keywords:** value-based, healthcare, cancer, public health, patient centeredness

Value in Healthcare, defined as the relationship between outcomes and costs (1), including direct financial costs and indirect costs such as impact on employment, treatment toxicity, and family/caregiver impact, remains a complex and multifaceted concept. Value-based healthcare is of primary importance in oncology as, over the past decades, neoplastic diseases have increased in incidence and prevalence, becoming one of the leading causes of death.

The objective of this Research Topic was to contribute to the existing body of knowledge with a clear picture of the current scenario of the Value-Based approach in oncologic clinical practice to deliver sound experience on its impact, potential benefits, challenges to address, and future research needs.

A total of 21 manuscripts were submitted, eleven of which were accepted.

Most of the contributions aimed at evaluating the economic efficacy or the financial impact of single/combined treatments of cancer diseases with a particular public health burden.

In other cases, authors shared the results of their research on the impacts of the application of effective models of value-based care (Bigi et al.); they analyzed a new tool to combine reported quality experiences and patient-reported outcomes (de Mattia et al.).

Also, organizational and financing implications from reshaping the delivery of healthcare services according to a Value-Based care approach have been published. For example, studies on the experience of involving patients and professionals in a co-constructed therapeutic pathway (Casà et al.); a Value-Based approach to untangle the full benefit of HPV-related cancers elimination strategies and identify priority and best practices (Calabrò et al.) have been reported, respectively.

*Value-based healthcare in oncology* still has some limits, as there remains no standard to quantify the many outcomes and cost components of value (i.e., patient-reported outcomes and estimated costs to patients), thus various conceptual frameworks have been proposed (2).

Furthermore, publishing has become more and more challenging and there are several reasons for the difficulties. I have encountered as an Editor that as a Public Health journal we cannot simply store papers that arrive spontaneously (even after a qualitative selection), and we tried to orient publishing according to validity, rigor, and relevance (3).

We also tried to challenge the public health research community to address future research needs, such as refinement of performance indicators to include patients' perspective (PROMS/PREMS); implementation of shared decision-making as routine in clinical practice; reshaping of logistics and operations to respect the values of “green” care delivery; digital support for the implementation of Value-Based approaches; reshaping of reimbursement systems to bundled payments based on clinical outcomes.

Indeed, we received a few feedbacks on some of these items.

Thus, from a public health perspective and by considering the great expansion of the research community, particularly from emerging countries and low-income countries, it will be crucial for healthcare organizations to use *Value-based healthcare in oncology* to improve the quality of care on an individual level and consider patients' concerns and needs; reduce unwarranted duplications and wastes in care provision via more regular or systematic assessment of the effectiveness of care and monitoring of disease progression; increase patient information, communication, and shared medical decision-making, thus paving the way for precision and personalized medicine.

## Author contributions

AB: Writing—original draft, Writing—review and editing.
